# Remarks on Dentistry in the Army

**Published:** 1861-09

**Authors:** Wm. B. Roberts

**Affiliations:** New York


Remarks on Dentistry in the Army.—By *Wm. B. Roberts, M. D.*, of New York.—The sad misfortunes of the Crimean war, owing to imperfect attention to the sanitary condition of the British army, opened the eyes of the whole civilized world to the importance of a proper sense of duty in this connection. It is not surprising, therefore, that humane and far-seeing men in this country should have taken an early opportunity in the present campaign, to press upon the government the almost incalculable importance of great care in the superintendence of the sanitary department of the army. Thus every effort is being made to attach to each regiment all the requisite force with all the necessary equipments to perform the work given to them in an efficient and satisfacdum, distributed to the parts which names it. The inferior maxillary, emerging through the oval foramen, subdividing into the lingual branch, which goes to the tongue, and into a motor, which supplies the muscles of mastication. The inferior maxillary nerve supplies the lower jaw and teeth. Neuralgic trismus is explainable by the compound nature of the nerve.

tory manner. There is probably very little to be said in the way of advice to such gentlemen as are thoroughly imbued with the importance of the subject, and as thoroughly acquainted with all the means and appliances necessary to it; but there is one point which has apparently escaped the attention of persons in authority, on which we wish to say a few words. During the last twenty years, and owing principally to the efforts of our own countrymen, *Dentistry* has become elevated from a mere branch to a distinct science. Colleges have been founded, societies formed, conventions held, professorships endowed, and all to give the greatest insight possible into the structure, physiology und pathology of the human teeth. It has been found, as men progressed in the study of these organs, that their disease spread its ramifications through the whole animal economy; all the functions of the body have been affected and indeed suspended by the existence of a foreign and improper condition in the teeth; the whole sanitary condition has been altered, and the most serious complications of disease have attended the existence of diseased teeth.

Thus dentistry has been taken out of the hands of the surgeons and doctors, and has formed a separate profession of enlightened, eucated, and scientific men, understanding as a necessity not only their own immediate calling, but also no small share of surgery and pathological anatomy. A few years since the question was raised in England as to the propriety of attaching dentists to the British army and navy; a great deal had been said upon the subject, and it was well known that the teeth of the soldiers and sailors were in a deplorable condition; but after a temporary prospect of success the subject was dropped. Meanwhile, in the French army, with that sense of decency that always characterizes the French nation, means had been taken to extend a proper attention to the wants of the body, and to the teeth; the men were supplied with brushes, and compelled to keep their teeth cleansed; and thus the matter stands in Europe at the present time. We have referred to this subject at the present time because it seems to us that it is proper that here, where dentistry has progressed more than in any other country, it is right that the first steps should be taken to still further advance it. And we desire especially to show the value that it might possess as a sanitary instrument, if proper efforts were made to invest it with the importance it deserves. It is almost needless to refer to the necessity for having good teeth in our army and navy; first, for mastication; and second, but most important, for their effect upon the general health; and upon this last subject we desire to give some information.

Careful mastication is essential to perfect digestion, and this latter, more especially in the active life of the soldier or sailor, is a primal necessity for healthy existence.

Very few persons are aware of the derangements which may be produced by diseased teeth, although the period of dentition in children is generally considered as one of the most critical in life. It is, indeed, estimated by some writers that one-tenth of all the deaths in the world occurs during the period of the first dentition. The animal frame is in infancy so delicate, that the least local irritation produces a sudden and universal sympathy throughout the whole body. Fever is a very frequent accompaniment of teething; also an affection of the skin, resembling measles; pustules sometimes appear, not unlike a mild form of small-pox; diseases of the scalp; diarrhea; convulsions; diseases of the lungs; and, in fact, symptoms of nearly every form of disease may be met with during the period, and resulting from dentition. If we know that this terrible catalogue of evils is attendant upon the growth of the teeth during infancy, is it not rational to suppose that a healthy condition of these organs is essential to adults, when we consider the close connection existing between the teeth, alveolar process, the parotid submaxillary and sublingual gland, on the one hand, and the mucous membrane of the mouth, which is continuous with that which lines the pharynx, œsophagus, stomach, and intestines, on the other?

It is well known to the dental practitioner, the physician and surgeon, that if a tooth becomes diseased, all these organs will to a certain extent sympathize with it, independent of the agonizing and excruciating pain caused by the exposure of the nerve. Cases have been given where inflammation of the mucous membrane has extended so far as to produce consumption; while dyspepsia with all its attendant horrors may be, and often is caused by improper and insufficient mastication, which must necessarily result from the possession of poor and imperfect teeth. Neuralgia and *tic-douloureux*, probably the most fearful pains which the human race ever suffers, often proceed in the first instance from exposed nerves; and other acute and chronic inflammatory diseases frequently spring from carious teeth and diseased gums, ruining health, and disabling their victims from performing even the most simple duties of life; and in the case of the soldier who is exposed night and day to every variety of inclement weather, and every quality of fare, the chances of such diseases establishing themselves through the means of diseased teeth are greatly multiplied.

It is well known to the dental profession that all the diseases common to teeth can not only be cured, but may be prevented by proper and timely treatment. After having thus enumerated the evils appertaining to a defective condition of the teeth, a condition which experience has found to exist among the army to an extraordinary extent, and having shown the importance of such diseased condition being avoided and changed to aid in establishing a proper sanitary condition among these men who risk their lives for their country's service, and have neither time, means, nor opportunity for themselves discharging their duty in this respect; we desire to urge upon the proper authorities, that a corps of dentists, or dental staff, should be attached to the United States army, similarly organized with the surgical department, who would act in connection with, and as an efficient aid to, that department, besides performing their own duties in a proper manner. Holding this to be a great sanitary measure, as well as an economical and humane movement in behalf of those affected by it, we would especially offer these suggestions to our "Sanitary Commission," believing that in the scope of their noble field of labor there could not be performed a more important act, than the procuring of the passage of a bill through Congress at the approaching session, which should incorporate into the army an efficient DENTAL staff, strengthened with all the powers necessary to enable it to become most serviceable to the cause of health.—*American Medical Times.*

